# Genetic analysis of the molecular regulation of electric fields-guided glia migration

**DOI:** 10.1038/s41598-020-74085-x

**Published:** 2020-10-08

**Authors:** Li Yao, Teresa Shippy, Yongchao Li

**Affiliations:** 1 Department of Biological Sciences, Wichita State University, 1845 Fairmount Street, Wichita, KS 67260 USA; 2 Bioinformatics Specialist, KSU Bioinformatics Center, Kansas State University, Manhattan, KS 66506 USA

**Keywords:** Biophysics, Cell biology, Computational biology and bioinformatics, Genetics, Physiology

## Abstract

In a developing nervous system, endogenous electric field (EF) influence embryonic growth. We reported the EF-directed migration of both rat Schwann cells (SCs) and oligodendrocyte precursor cells (OPCs) and explored the molecular mechanism using RNA-sequencing assay. However, previous studies revealed the differentially expressed genes (DEGs) associated with EF-guided migration of SCs or OPCs alone. In this study, we performed joint differential expression analysis on the RNA-sequencing data from both cell types. We report a number of significantly enriched gene ontology (GO) terms that are related to the cytoskeleton, cell adhesion, and cell migration. Of the DEGs associated with these terms, nine up-regulated DEGs and 32 down-regulated DEGs showed the same direction of effect in both SCs and OPCs stimulated with EFs, while the remaining DEGs responded differently. Thus, our study reveals the similarities and differences in gene expression and cell migration regulation of different glial cell types in response to EF stimulation.

## Introduction

In a developing nervous system, endogenous electric fields (EFs) affect embryonic growth^1–4^. Interestingly, EF vectors seem to be aligned with major embryonic axes^5^. Additionally, electrical activity regulates the path-finding of growing axons and the formation of initial connections in the developing nervous system. For example, studies have reported the influences of electrical activity on the projection of growing thalamic axons towards their appropriate cortical target area^6^ and the formation of layer-specific connections by axons of cortical pyramidal neurons ^7^.

Neural trauma causes neural cell necrosis, demyelination, and degeneration of axons, which lead to functional loss. Glial cell transplantation may enhance remyelination at the lesion. Oligodendrocytes and Schwann cells (SCs) are glial cells that myelinate axons in the central nervous system (CNS) and in the peripheral nervous system (PNS), respectively^8–10^. In post-spinal cord injury, the movement of oligodendrocyte precursor cells (OPCs) from gray and white matter to the lesion helps re-myelinate the axons^11,12^. Schwann cells can migrate to the injury lesion to myelinate spinal axons^13,14^. Both SCs and OPCs have been transplanted into wounded spinal cords, and the implantation of these cells enhanced spinal axonal regeneration^15–17^.

The effect of EFs on cell migration has been reported in a number of investigations. In previous studies, we found that both SCs^18^ and OPCs migrated toward the anodal pole of an applied EF^19^. The increase of directedness of anodal migration of these cells is in proportion to the strength of the applied EF (50 to 200 mV/mm). During directed cell migration, cells become polarized dynamically in response to guidance signals. The polarized cell shape is characterized by membrane ruffling and filopodia at the front edge^20^. The guided cell movement requires coordinated reorganization of the actin cytoskeleton and microtubules (MTs) in the leading and trailing processes and cell body. However, the molecular pathways regulating this critical process have not been elucidated.

To understand the signaling molecules that regulate the EF-guided migration of Schwann cells and OPCs, next-generation RNA sequencing (RNA-seq) was performed to identify the genes that play important roles in regulating directional cell migration^18,19^. In those studies, we determined the differentially expressed genes (DEGs) for SCs and OPCs subjected to EF stimulation. The RNA-seq assay of SCs identified 1045 up-regulated and 1636 down-regulated genes in cells with EF stimulation (100 mV/mm) versus control cells. A Kyoto Encyclopedia of Genes and Genomes (KEGG) pathway assay showed that 21 pathways are down-regulated, while 10 pathways are up-regulated for EF-stimulated cells compared with control cells. Several cellular signaling pathways that are involved in the regulation of cell migration, including pathways regulating the actin cytoskeleton, focal adhesion, and PI3K-Akt were recognized. The RNA-seq assay of OPCs revealed that the mitogen-activated protein kinase (MAPK) pathway that signals cell migration was significantly up-regulated in EF-stimulated cells compared with control cells. Gene ontology (GO) enrichment analysis showed an enrichment of chemotaxis-related GO terms among down-regulated DEGs.

These studies revealed the differentially expressed genes that regulate the EF-guided migration of SCs or OPCs alone. However, the major similarities and differences in how EFs affect gene expression in different cell types have not been studied. In this study, we analyzed the DEGs in these two studies jointly and explored their function using GO terms, focusing on the cytoskeleton, cell adhesion, cell movement, and cell migration. Our results give insight into similarities and differences in DEGs and cell migration regulation of different glial cell types in response to EF stimulation.

## Results

### RSEM results

To better understand the similarities and differences in the response of Schwann cells and OPCs to an EF, we performed meta-analysis based on previously described RNA-seq data from two studies^18,19^. Four conditions were included in the analysis: EF-stimulated OPCs, control OPCs, EF-stimulated Schwann cells, and control Schwann cells. We identified 9364 genes that showed differential expression in at least one of these conditions. Of these DEGs, 1907 showed differential expression in at least one of the EF conditions and were sorted into eight categories based on the direction of expression change, if any, in each cell type (Supplemental Table 1), as follows: (1) EF-induced gene down-regulation in both OPCs and SCs (Both Down); (2) EF-induced gene up-regulation in both OPCs and SCs (Both Up); (3) EF-induced gene down-regulation in SCs and no change in OPCs (SC-Down/OPC-No Change); (4) EF-induced gene up-regulation in SCs and no change in OPCs (SC-Up/OPC-No Change); (5) EF-induced gene down-regulation in OPCs and no change in SCs (OPC-Down/SC-No Change); (6) EF-induced gene up-regulation in OPCs and no change in SCs (OPC-Up/SC-No Change); (7) EF-induced gene up-regulation in SCs and gene down-regulation in OPCs (SC-Up/OPC-Down); and (8) EF-induced gene up-regulation in OPCs and gene down-regulation in SCs (OPC-Up/SC-Down).

**Table 1:** DEGs associated with GO terms related to cytoskeleton, cell adhesion, cell movement, and cell migration.

Category	GO term	Gene number	Gene name
Both down	CC cytoskeleton	32	Apc2, Cdc42ep4, Dlgap5, Dsn1, Gtse1, Lasp1, Nckap5l, Sh2b2, Taok2, Tpx2, Triobp, Cdc7, Cep250, Cdk2, Ckap2, Dgkq, Espl1, Flnc, Fhod1, Klhl12, Klhl22, Kif20a, Kif2c, Mark4, Mcm2, Map3k11, Notch1, Nusap1, Plekhh3, Spag5, Timeless, Zw10
Both up	MF focal adhesion	9	ADP-ribosylation factor 6(Arf6), Rho family GTPase 3(Rnd3), calponin 3(Cnn3), cortactin(Cttn), melanoma cell adhesion molecule(Mcam), plasminogen activator urokinase receptor(Plaur), poliovirus receptor(PVR), testin LIM domain protein(Tes), zyxin(Zyx)
OPC-down/SC-no change	CC cytoskeleton	63	Mtrr, Abl1, Bcar1, Clip2, Dennd2a, Dnaja3, Mdm1, Pms2, Pxk, Rimbp3, Racgap1, Src, Ssx2ip, Tbccd1, Xrcc2, Aif1l, Amot, Aurkb, Ambra1, Cenpe, Cenpf, Cenpj, Cep164, Cep55, Cep72, Ccnb1, Ccnf, Flna, Flnb, Gja1, Incenp, Ift80, Kif18b, Kif1c, Kif23, Kif4a, Kifap3, Knstrn, Lmnb1, Lmnb2, Lcp1, Mark2, Map4, Myo1c, Nphp4, Nf2, Nos2, Pik3r4, Plk1, Prc1, Ralbp1, Specc1l, Spdl1, Sybu, Ttc8, Top2a, Tmem214, Tsc2, LOC100909441, Tubd1, Tube1, Tubgcp2, Tubgcp5
SC-up/OPC-no change	BP cell adhesion	62	Axl, Bcl2, Cd24, Cd44, Cited2, Ehd4, Ets1, L1cam, Lims2, Mb21d2, Pdlim1, Prdm1, Smad6, Actn4, Actn1, Ap3d1, Ajap1, Adgrg1, Bcan, Csnk1d, Cadm3, Cadm4, Col18a1, Ctgf, Cyp1b1, Dusp3, Epdr1, Efnb2, Ezr, Fermt2, Flrt2, Fbln2, Fstl3, Foxp1, Gcnt2, Hspa5, Hnrnpk, Has2, Il6st, Lama4, Lamc2, Lef1, Map2k1, Myh9l1, Nectin3, Nrg1, Parvb, Pxn, Postn, Pkp1, P2ry12, Ripk2, Runx2, Serpine1, Sdc4, Tspan5, Thbs1, Thbs2, Tinagl1, Tnfsf18, Vegfc, Vasn
	BP cell migration	53	Arf4, Axl, Bcl2, Ccl22, Cd24, Cd44, Cited2, Ets1, Gata2, Sh3kbp1, Stard13, Abhd6, Actn4, Adgrg1, Clic4, Col18a1, Ctgf, Cyp1b1, Efnb2, Fgf2, Fgf7, Flrt2, Foxp1, Gdnf, Gcnt2, Hspa5, Hbegf, Has2, Igfbp3, Il6st, Lamc2, Lef1, Map2k1, Myh9l1, Nrg1, Nfe2l2, Ndel1, Postn, Plat, Pdgfb, Plxnd1, Pkn3, P2ry12, Serpine1, Shtn1, Sphk1, Sdc4, Thbs1, Tfap2a, Tnfsf18, Twist1, Tyk2, Vegfc
	CC focal adhesion	29	Akap12, Cd44, G3bp1, L1cam, Lims2, Pdlim1, Rab21, Arhgap22, Sh3kbp1, Actn4, Actn1, Efnb2, Ezr, Fermt2, Flrt2, Fzd2, Hspa5, Hnrnpk, Hmga1, Map2k1, Mprip, Myh9l1, Parvb, Pxn, Pabpc1, Pcbp2, Procr, LOC100360679, Sdc4
SC-down/OPC-no change	BP locomotion	49	Asap3, Cxcl6, Ephb4, Gli2, Ldb1, Six4, Slc9a3r1, Vangl2, Ank3, Aqp1, Celsr2, Col1a1, Col5a1, Csf1, Dst, Efnb1, Efnb3, Emp2, Erbb2, Epb41l4b, Fgf10, Foxo4, Hgf, Itga11, Itga4, Itgb4, Kif26b, Lama2, Lama5, Lamb1, Lpar1, Matn2, Mmp2, Msx2, Nefl, Nrp1, Nr2f2, Plekhg5, Pawr, Rreb1, Reck, Rffl, Sfrp2, Sema3b, Sema3g, Stat5a, Snai2, Tns1, Tgfbr3
	BP regulation of cell adhesion	26	Cd59, Ephb4, Lmo7, Ldb1, Ank3, Celsr2, Col1a1, Csf1, Efnb1, Efnb3, Emp2, Erbb2, Erbb3, Epb41l4b, Itga4, Kif26b, Lama2, Lama5, Lamb1, Mmp2, Nid1, Pawr, Rreb1, Stat5a, Snai2, Utrn

### Analysis of DEGs and GO analysis for OPCs and SCs subjected to EF stimulation

We performed GO term enrichment analysis on the DEGs in each of the eight categories. Gene ontology analysis provides information on the biological process (BP), molecular function (MF) and cellular component (CC) associated with particular genes. The significantly enriched GO terms of various DEGs in these categories were identified. GO terms that are related to the cytoskeleton, and adhesion and migration in the BP, MF, and CC classifications were of particular interest.

**Figure 1:**
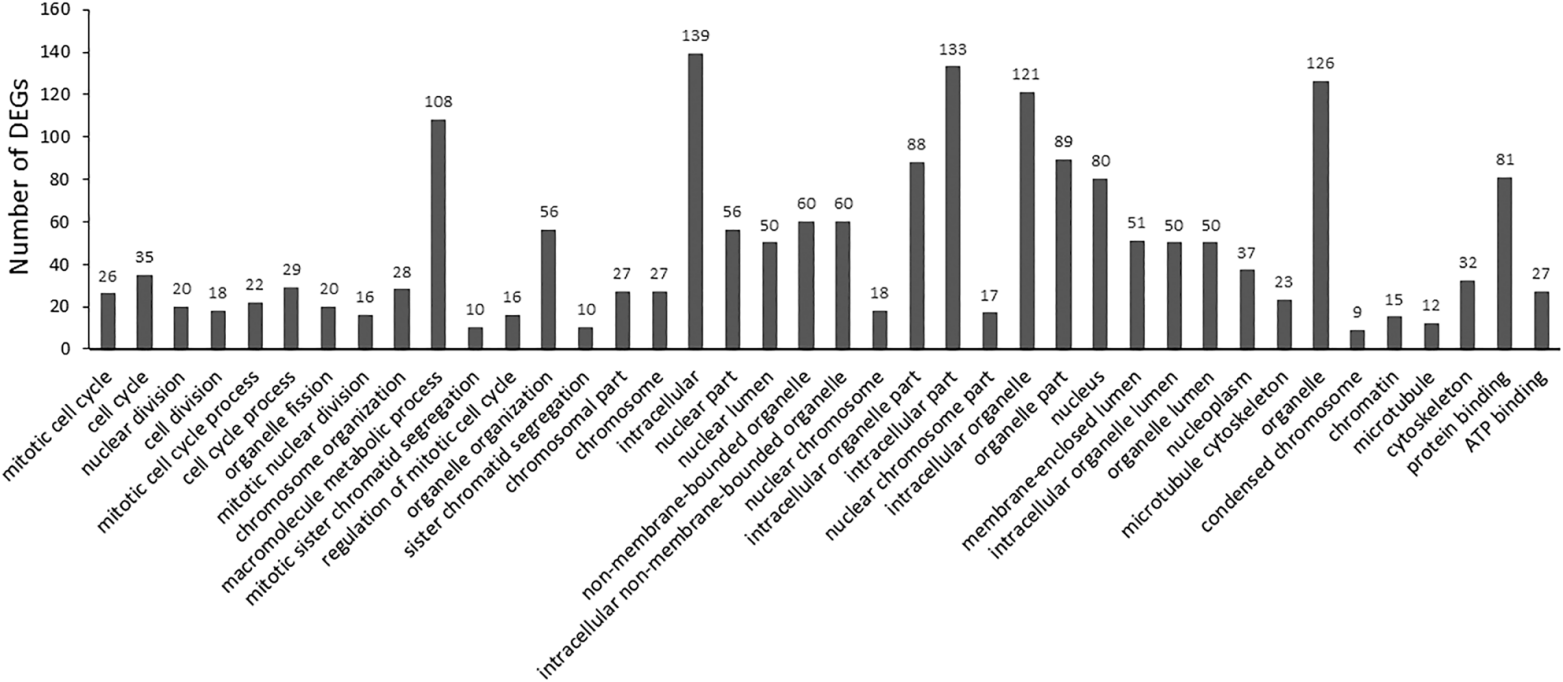
EF-induced gene down-regulation in both OPCs and SCs (both down).

In the category of Both Down (Fig. 1), 193 DEGs of down-regulated gene were identified. In this category, the significantly enriched BP includes 14 terms, which are cell cycle and cell division-related terms, including mitotic cell cycle, cell division, and mitotic sister chromatid segregation. The significantly enriched CC terms (n = 24) are chromosome and intracellular-related terms as well as terms related to the cytoskeleton. The significantly enriched MF terms are protein binding and ATP binding.

**Figure 2:**
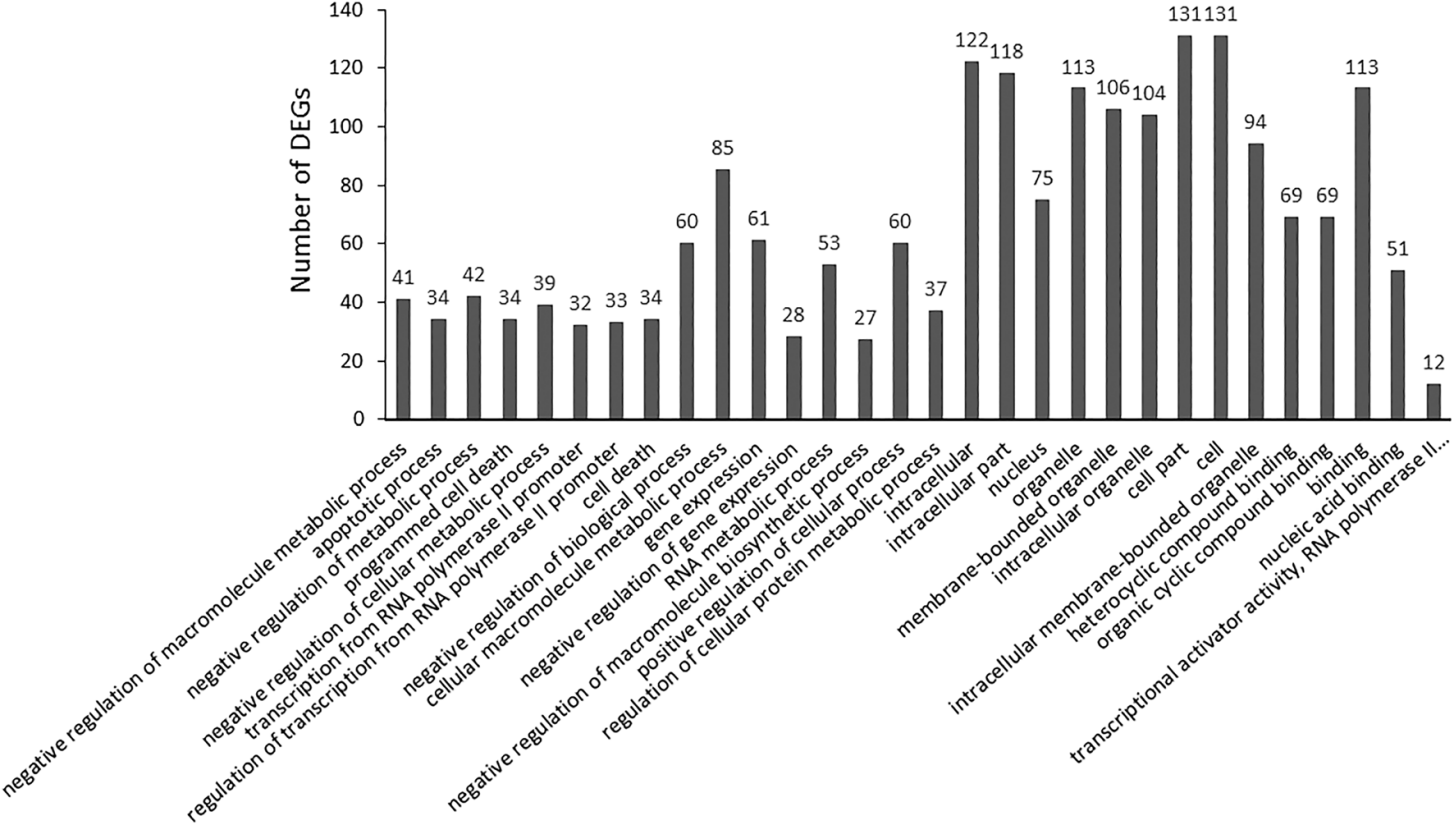
EF-induced gene up-regulation in both OPCs and SCs (both up).

In the category of Both Up (Fig. 2), 154 DEGs were identified. The significantly enriched BP terms (n = 16) are negative regulation of macromolecule metabolic process, apoptotic process, and transcription from RNA polymerase II promoter-related terms. The significantly enriched CCs are intracellular, nucleus, and organelle-related terms (n = 9). Nine significantly up-regulated genes (Table 1) associated with focal adhesion (p = 0.9) were recognized, although this it is not a significantly enriched term. The significantly enriched MF terms are heterocyclic compound binding, organic cyclic compound binding, binding, nucleic acid binding transcriptional activator activity, and RNA polymerase II transcription regulatory region sequence-specific binding.

**Figure 3:**
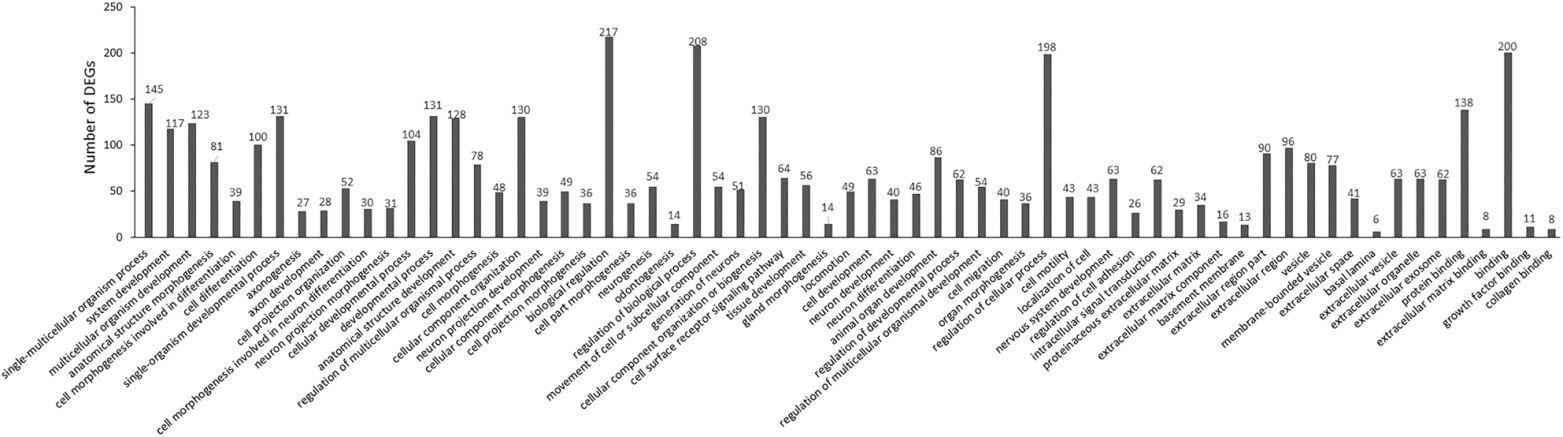
EF-induced gene down-regulation in SCs and no change in OPCs (SC-down/OPC-NO change).

In the category of SC-Down/OPC-No Change (Fig. 3), 313 DEGs were identified. The significantly enriched BP terms (n = 47) include the single-multicellular organism process, system development, multicellular organism development, cell differentiation, and related terms. The significantly enriched CC terms (n = 13) include extracellular matrix, extracellular vesicle, and related terms. The significantly enriched MF terms (n = 5) include protein binding, extracellular matrix binding, binding, growth factor binding, and collagen binding.

**Figure 4:**
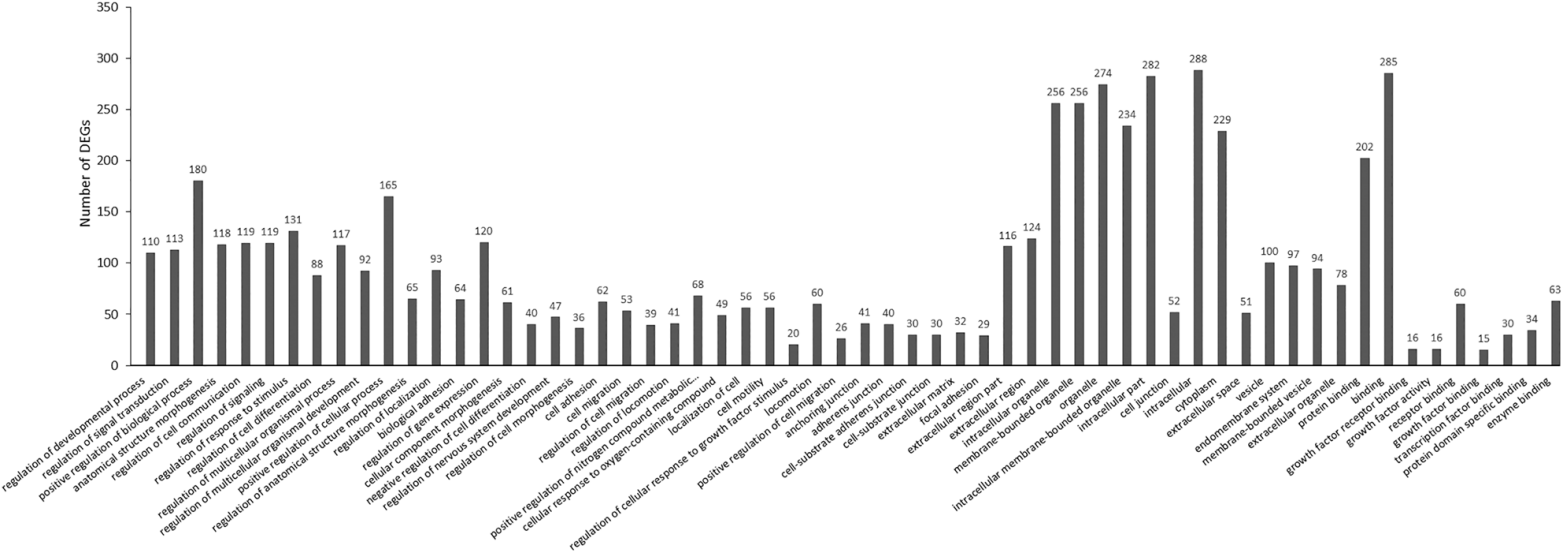
EF-induced gene up-regulation in SCs and no change in OPCs (SC-Up/OPC-no change).

In the category of SC-Up/OPC-No Change (Fig. 4), 410 DEGs were identified. The significantly enriched BP terms (n = 256) include regulation of developmental process, regulation of signal transduction, positive regulation of biological process, anatomical structure morphogenesis, and related terms. The significantly enriched CC terms (n = 21) include adherens junction, cell-substrate adherens junction, cell-substrate junction, extracellular matrix, focal adhesion, and related terms. The significantly enriched MF terms (n = 9) include protein binding, growth factor, receptor binding, growth factor activity, receptor binding, growth factor binding, transcription factor binding, protein domain specific binding, and enzyme binding.

**Figure 5:**
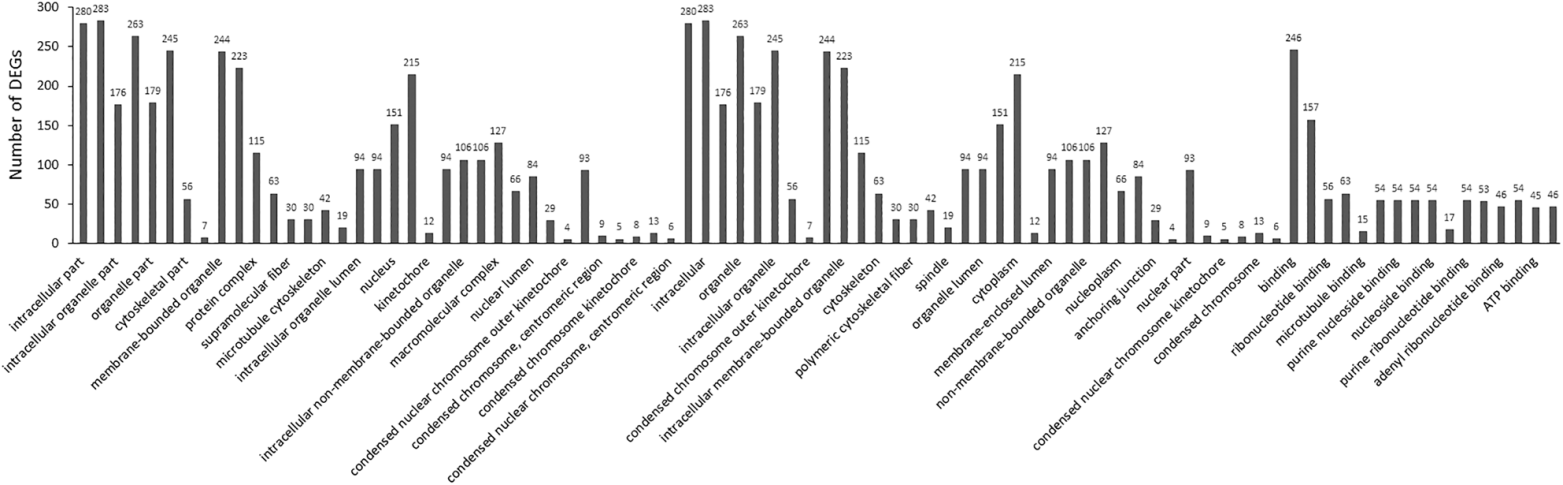
EF-induced gene down-regulation in OPCs and no change in SCs (OPC-down/SC-no change).

In the category of OPC-Down/SC-No Change (Fig. 5), 402 DEGs were identified. The significantly enriched BP terms (n = 20) include cell cycle, sister chromatid segregation, positive regulation of cellular process, and related terms. The microtubule-based process and microtubule cytoskeleton organization are also enriched terms. The significantly enriched CC terms (n = 35) include intracellular, organelle, nucleus, and related terms. The cytoskeletal part, cytoskeleton, and polymeric cytoskeletal fiber are also significantly enriched terms in this category. The significantly enriched MF terms (n = 16) include protein binding, ribonucleotide binding, carbohydrate derivative binding, and microtubule binding.

**Figure 6:**
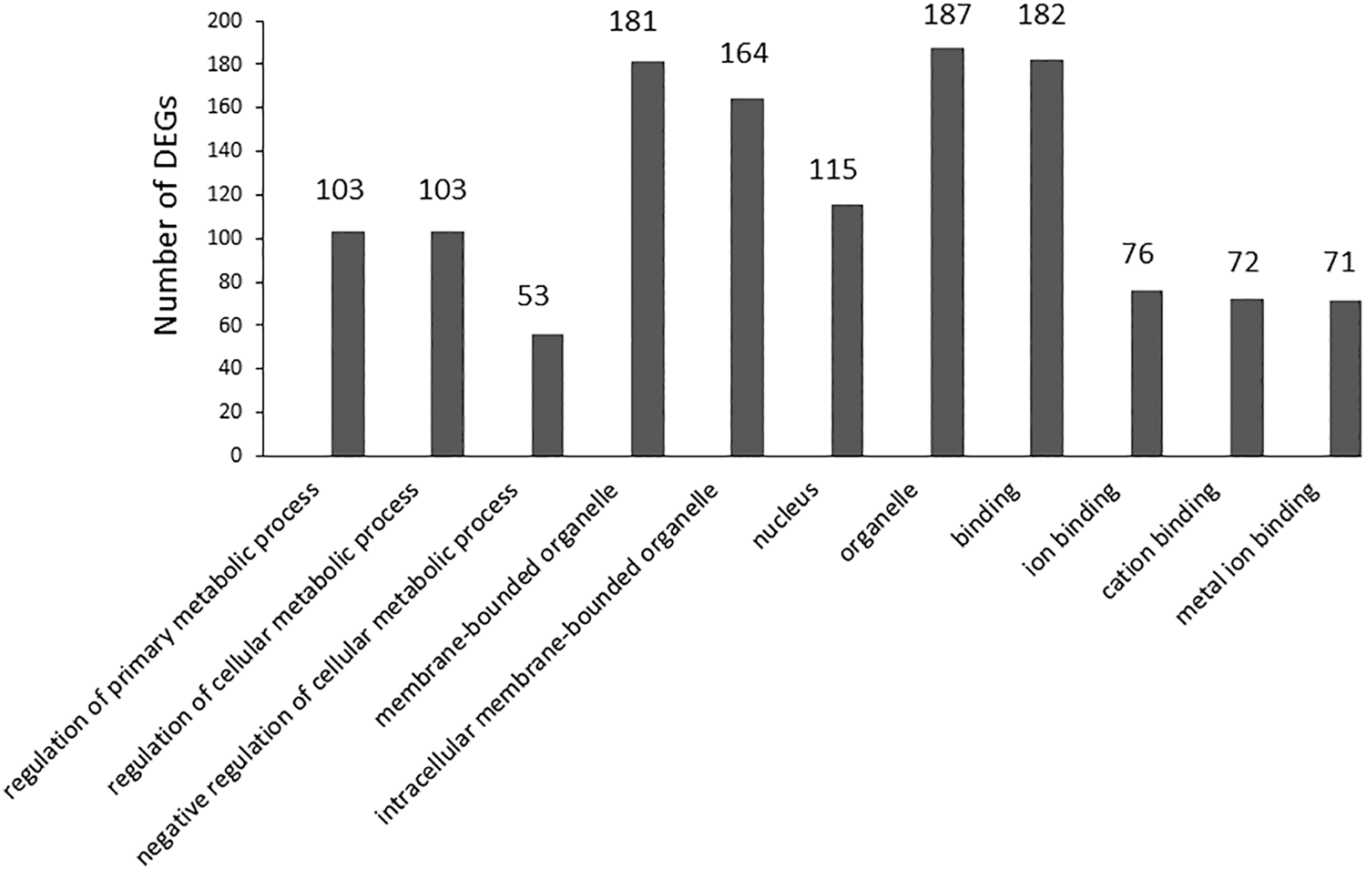
EF-induced gene up-regulation in OPCs and no change in SCs (OPC-up/SC-no change).

In the category of OPC-Up/SC-No Change (Fig. 6), 341 DEGs were identified. The significantly enriched BP terms (n = 3) include regulation of the primary metabolic process, regulation of the cellular metabolic process, and negative regulation of the cellular metabolic process. The significantly enriched CC terms (n = 4) include membrane-bounded organelle, intracellular membrane-bounded organelle, nucleus, organelle. The significantly enriched MF terms (n = 4) include binding, ion binding, cation binding, and metal ion binding.

**Figure 7:**
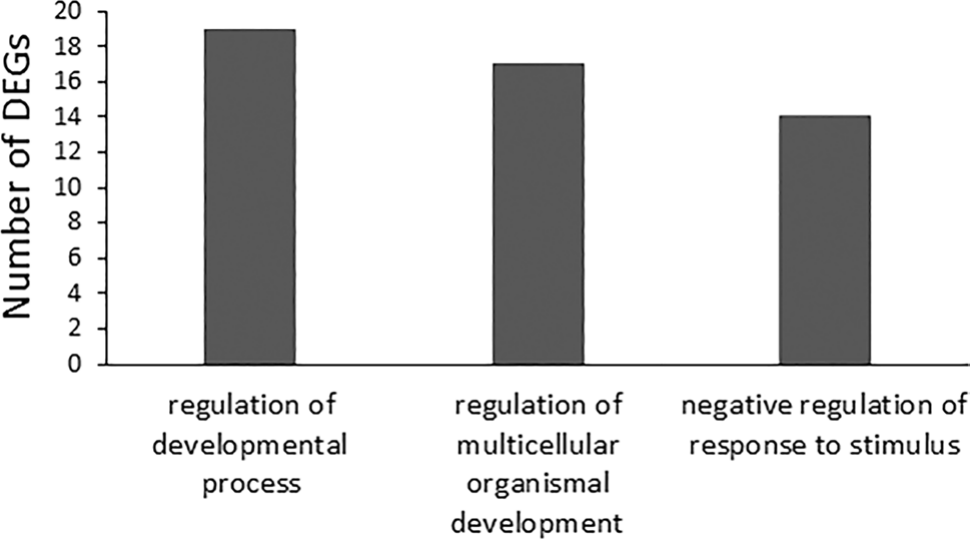
EF-induced gene up-regulation in SCs and down regulation in OPCs (SC-up/OPC-down).

In the category of SC-Up/OPC-Down (Fig. 7), 52 DEGs were identified. The significantly enriched BP terms (n = 3) include regulation of developmental process, regulation of multicellular organismal development, and negative regulation of response to stimulus. We did not recognize any significantly enriched terms in CC and MF.

In the category of OPC-Up/SC-Down, 42 DEGs were identified. We did not recognize any significantly enriched terms in BP, CC, and MF.

### DEGs regulating cytoskeleton, adhesion, and migration

The cytoskeleton of Schwann cells and OPCs are organized by genes that regulate cell adhesion and migration. The DEGs associated with significantly enriched GO terms involving cytoskeleton, cell adhesion, or cell movement and migration are summarized in Table 1. In the category of Both Down, the significantly enriched CC-type GO term—cytoskeleton—includes 32 DEGs. In the category of OPC-Down/SC-No Change, the significantly enriched CC-type GO term—cytoskeleton—includes 63 DEGs. In the category of SC-Down/OPC-No Change, the significantly enriched BP-type GO terms—locomotion and cell motility—include 49 and 26 DEGs, respectively. In the category of SC-Up/OPC-No Change, the significantly enriched BP-type GO terms—cell adhesion and cell migration—include 62 and 53 DEGs, respectively. In this category, the significantly enriched CC-type GO term—focal adhesion—includes 29 DEGs.

## Discussion

During directed cell migration, cells become polarized dynamically in response to guidance signals. The polarized cell shape is characterized by membrane ruffling and filopodia at the front edge, reorientation of the microtubule-organizing center (MTOC) and the Golgi apparatus in the direction of migration, and coordinated reorganization of the actin cytoskeleton and microtubules in the leading process^21,22^. In focal adhesions, F-actin and other associated molecules form a structure that enables cells to adhere to the extracellular matrix^21,22^. Signal transduction is involved in the assembly and disassembly of the protein complex thereby controlling cell motility and migration direction. In previous publications, we have reported that the migration of SCs and OPCs can be directed by an applied electric field, and we performed RNA-seq to explore the pathways that regulated the cell migration for each type^18,19^.

In this study, we jointly analyzed the RNA-seq data for OPCs and SCs subjected to EFs and then identified significantly enriched GO terms associated with DEGs. We were particularly interested in the significantly enriched GO terms related to cytoskeleton, cell adhesion, cell movement, and cell migration. Below we discuss some of the DEGs associated with these terms with respect to the direction of regulation in each glial cell type. Effective directional migration of OPCs and Schwann cells to a neural lesion is crucial in the neural remyelination process. Potentially an applied EF can guide the cells to migrate into the lesion to enhance the remyelination of axons. Our studies provide clues to understand the gene regulation of cell migration. To explore the gene expression for the neural cells subjected to EF stimulation may also lead to the improvement of strategies to regulate neural cell migration.

### Both down: cytoskeleton (CC)

The organization of cytoskeleton controls cell migration. Previous studies showed that a number of DEGs that were down-regulated in both SC and OPCs subjected to EF stimulation regulate cytoskeleton function and cell migration. The TRIOBP gene is a member of the Rho guanine nucleotide exchange factor. It regulates cell spreading and cell contraction by organizing actin formation; therefore, it is involved in cell motility^23,24^. LIM and SH3 protein 1 (LASP1) binds to the actin cytoskeleton at the cell membrane extension and regulates the dynamic actin-based cell adhesion and motility. It was reported that overexpression of LASP1 is associated with proliferation, migration, and invasion in esophageal squamous cell carcinoma^25^. The FORMIN homology 2 domain containing protein 1 (FHOD1) is required for the assembly of F-actin structures, such as stress fibers. The Rho-ROCK signaling pathway regulates its function. One study of mouse fibroblast migration showed that FHOD1 is recruited to integrin clusters that lead to actin assembly^26^. That study suggested that FHOD1 is needed for directed forces and cell adhesion during cell spreading and migration. The CDC42 effector protein 4 (CDC42EP4) is a CDC42-binding protein that interacts with CDC42 to induce actin filament assembly, which regulates cell shape and movement^27^. A previous study showed that overexpression of PAK4, a CDC42-activated kinase that decreases adhesion, enhanced galvanotaxis speed of fibroblast^28^. However, we observed the decreased expression of CDC42EP4. Further investigation will help to explore the regulation of CDC42 and its effector proteins. The APC regulator of WNT signaling pathway 2 (APC2) regulates actin assembly and microtubule network formation, and therefore regulate cell adhesion and motility. The down-regulation of these genes in both SCs and OPCs subjected to EF stimulation suggests a similar function of these genes for the regulation of both cell types.

### Both up: focal adhesion (CC)

Although focal adhesion is not a significantly enriched term for the Both Up category, we summarized the function of nine significantly changed genes associated with this term. A number of studies have reported the role of small GTPase family members such as Rho^29^ and Rock^30^ in the regulation of EF-directed cell migration. In general, inhibition of these molecules abolished the EF-directed cell migration^29,30^. This study indicated the function of an additional GTPase member in EF-directed cell migration. The Rho family GTPase 3 (RND3) is a member of a small GTPase family. The protein acts as a negative regulator of fibroblast cytoskeletal organization that can lead to loss of adhesion^31^. Cortactin (CTTN) regulates the organization of the actin cytoskeleton and cell shape in processes such as lamellipodia formation. The cortactin N-terminal half binds and activates the ARP2/3 complex and therefore regulates actin dynamics. It was reported that overexpression of either full-length cortactin or the cortactin C-terminus can enhance the migration of mammary epithelial cells^32^. In a previous study, we reported the cathodal migration of neural stem cells–derived OPCs subjected to EF stimulation. The ARPC2^−^/^−^ OPCs lost the directional migration in EF^33^. This study indicated that the function of ARPC2/3 complex and the activation of ARPC2/3 complex are required for EF-directed cell migration. The plasminogen activator, urokinase receptor (PLAUR), mediates the proteolysis-independent signal transduction activation effects of U-PA. The inhibition of PLAUR decreases the growth, invasion, angiogenesis, and metastasis of various cancers^34–36^. The ZYXIN (ZYX) protein concentrates at sites of focal adhesions and along the actin cytoskeleton in a cell. It was reported that in migrating epithelial cells, ZYXIN accumulates at force-bearing sites at the leading edge but not at the trailing edge. Rho-kinase and myosin II activation also regulated ZYXIN recruitment^37^. The result indicated that these up-regulated genes may be involved in the regulation of focal adhesion and migration of both SCs and OPCs subjected to EF stimulation.

### SC-down/OPC-no change: locomotion (BP)

The significantly enriched GO terms are the BP terms locomotion and regulation of cell adhesion. There are 49 DEGs associated with the term locomotion, ARFGAP with SH3 domain, Ankyrin Repeat, and PH domain 3 (ASAP3) is a focal adhesion-associated ARFGAP and regulates cell migration and focal adhesion. It was reported that the reduced expression of ASPS3 decreased cell motility of glioblastoma cells and cell invasion of mammary carcinoma cells^38^. The cadherin EGF LAG seven-pass G-type receptor 2 (CELSR2) is a protein that is involved in the cell matrix or cell-to-cell communication in cell adhesion. Based on the phenotypes of knockout mice, one study showed that the CELSR2 and 3 genes controlled the migration ability of facial branchiomotor neurons. Ephrin B1 is a type I membrane protein and a ligand of Eph-related receptor tyrosine kinases, which are crucial for migration, repulsion, and adhesion. The interaction of Eph receptors and ephrins can lead either to cell repulsion or cell adhesion and invasion^39^. The epithelial membrane protein 2 (EMP2) functions as a key regulator of cell membrane composition and promotes the recruitment of integrins to lipid rafts. A previous study showed that Emp2 regulates cell migration and cell adhesion. EMP2 governs transepithelial migration of neutrophils into the airspace^40^. MATRILIN 2 (MATN2) is a multiadhesion adaptor protein that interacts with other ECM proteins and integrins. MATN2 promotes neurite outgrowth and Schwann cell migration^41,42^. NEUROPILIN 1 (NRP1) regulates actin network organization through RhoA and ROCK. It was reported that SEMA3D controls endothelial cell migration by molecular mechanisms via NRP1 and PLXND1^43^.

### SC-down/OPC-no change: cell adhesion (BP)

The integrin subunit alpha 4 (ITGA4) associates with a beta 1 or beta 7 subunit to form an integrin that is involved in cell motility and migration. An in vitro study showed that ITGA4 overexpression on mesenchymal stem cells (MSCs) enhances transendothelial migration^44^. The results indicated the involvement of down-regulated genes in the regulation of SC migration in an applied EF but not in OPCs.

### SC-up/OPC-no change: cell adhesion (BP)

The AXL receptor tyrosine kinase (AXL) regulates the extracellular matrix protein expression and demonstrates that it is essential for invasion and metastasis of endometrial cancer. Studies have shown that AXL gene silencing inhibited the migration and invasion of endometrial cancer cells^45^. The L1 cell adhesion molecule (L1CAM) is an immunoglobulin superfamily protein that regulates neuronal migration and differentiation in nervous system development^46^. The adherens junctions associated protein 1 (AJAP1) is a type-I transmembrane protein that localizes and interacts with the E-cadherin-catenin complex. The endogenous AJAP1 is associated with the microtubule cytoskeleton and can attenuate sprouting angiogenesis by reducing endothelial migration and invasion capacities^47^.

### SC-up/OPC-no change: cell migration (BP)

Actinin alpha 4 (ACTN4) is an actin cross-linking protein that regulates cell protrusion I cell movement. ACTN4 promotes migration and metastasis of osteosarcoma through the NF-κB Pathway^48^. Inhibition of ACTN4 decreased the formation of cell filopodia and therefore suppressed tumor cell migration^49^. The connective tissue growth factor, also known as CCN2, mediates cell adhesion, aggregation, and migration in a few cell types, such as vascular endothelial cells, fibroblasts, and epithelial cells^50^. The NudE neurodevelopment protein 1-like 1 (NDEL1) regulates microtubules and intermediate filaments. NDEL1 regulates cell movement by interacting with TRIO-associated repeat on actin (TARA), which is an actin-bundling protein^51^.

### SC-up/OPC-no change: focal adhesion (BP)

PDZ and LIM domain 1 (PDLIM1) contains a C-terminal PDZ and one or more N-terminal LIM domain. It acts as an adapter that brings signal proteins to the cytoskeleton. It has been shown that the PDLIM protein CLP36 is critical for stress fiber formation and the assembly of focal adhesions in BeWo cells^52^. PDLIM1 overexpression attenuated the epithelial-mesenchymal transition of colorectal cancer cells^53^. Rho GTPase activating protein 22 (ARHGAP22) converts RAC1 to an inactive GDP-bound state that inhibits RAC1-dependent lamellipodium formation^54^. Forced expression of ARHGAP22 suppressed lamellae formation and cell spreading^55^. EZRIN (EZR) serves as an intermediate between the plasma membrane and the actin cytoskeleton, and regulates cell adhesion and migration. EZR overexpression causes the increased invasion of cancer cells^56,57^. The studies suggested that these up-regulated genes regulated Schwann cell migration in an EF. However, further studies will be required to determine how the EF regulates focal adhesion and therefore controls cell migration.

### OPC-down/SC-no change: cytoskeleton (CC)

Integrin is cell surface receptor regulates the function of the small GTPase Rac1 and therefore control the cell membrane protrusion and cytoskeletal reorganization events in directional migration. The Rac GTPase-activating protein 1 (GAP1) links β1 integrin to Rac1. A study showed that siRNA-mediated knockdown of either filamin-A or IQ-motif-containing GTPase-activating protein 1 (IQGAP1) induced high, dysregulated Rac1 activity during cell spreading on fibronectin^58^. Tyrosine kinase SRC and its downstream signaling molecules, including the small GTPase Rac1 and Arp2/3 complex, regulate the polymerization of actin network that initiates membrane protrusion in cell migration^59^. It was reported that the cell front of a migrating fibroblast was defined by integrin activation. The subsequent FAK, Src, and p190RhoGAP induces reorientation of the nucleus and the establishment of front–rear polarity^60^. Microtubule-associated protein 4 (MAP4) protein stabilizes microtubules by modulating microtubule dynamics. A previous study showed that MAP4 regulates invasion and migration of esophageal squamous cancer cells. MAP4 promotes cell invasion and migration by activating the ERK-c-Jun-vascular endothelial growth factor A signaling pathway^61^.

Effective directional migration of OPCs and Schwann cells to a neural lesion is crucial in the neural remyelination process. Potentially an applied EF can guide the cells to migrate into the lesion to enhance the remyelination of axons. Our studies provide clues to understand the gene regulation of cell migraiton. Exploring the gene expression in neural cells subjected to EF stimulation may also lead to the improvement of strategies to regulate neural cell migration.

## Conclusion

In summary, in this study, we performed comparative differential expression analysis for RNA-seq reads from OPCs and SCs that were subjected to EF stimulation in our previous reports in Yao et al.^18^ and Li et al.^19^, respectively. GO term analysis of the DEGs revealed a number of significantly enriched GO terms related to cytoskeleton, cell adhesion, cell movement, and cell migration. Of the DEGs associated with these terms, nine up-regulated DEGs and 32 down-regulated DEGs showed the same direction of effect in both SCs and OPCs stimulated with EFs, while the remaining DEGs showed different effects in SCs and OPCs subjected to EF stimulation. The DEGs revealed in this study support the findings in previous reports regarding the function of genes that regulated EF-directed cell migration, for example the expression of cortactin, an activator of the ARP2/3 complex, increased in both SC and PCs. Additionally, this study revealed that the genes that generally regulate cell adhesion and migration in some cases follow the same transcription trend during the regulation of EF-guided migration of different cell types, but in other cases show different transcriptional effects. This outcome suggests that some processes involved in the migration of different glial cell types are similar, but there are likely processes specific to each cell type as well. Although the DEGs that regulate cell migration are recognized in the GO term analysis, no signaling pathways show significant enrichment of DEGs. This suggests that existing established signaling pathways may not be the key regulators of EF-guided cell migration.

## Methods

The RNA-seq reads from OPCs and Schwann cells exposed to electrical fields were previously described in the work of Yao et al.^18^ and Li et al.^19^, respectively. The OPCs and Schwann cells that were used in those studies were isolated from cerebral cortexes of postnatal rats (days 1–2) and the sciatic nerves of postnatal rats (days 1–3) respectively. After culturing, OPCs or Schwann cells (100,000 cells) were then seeded in a cell culture chamber for EF stimulation. RNA was extracted for RNA sequencing studies after the cells were subjected to EF stimulation. The cells without EF stimulation were used as control. In this study, for our comparative analysis, we omitted the reads from OPCs exposed to 100 V, since only exposure to 200 mV showed a significant effect on cell migration. We also used only the R1 reads from the SCs, since the OPC reads were single-end reads. All reads were trimmed for adapter contamination and sequence quality with a custom pipeline using the adaptive trimming tools Scythe^62^, Trimmomatic-0.33^63^, and Sickle^64^. Differential expression results comparing four conditions (200 V OPCs, control OPCs, 100 mV SCs, and control SCs) were calculated using the RSEM-EBSeq pipeline^65,66^). First, reads were aligned to the Rnor_6.0 reference genome with Bowtie 2, and then gene-level counts from RSEM were used by EBSeq to calculate the probability of differential expression for each of 15 possible expression patterns (Supplemental Table 1). False discovery rate (FDR) cutoffs were applied to the results to generate lists of differentially expressed genes at less than 5 percent and 1 percent FDR (Supplemental Table 2).

The patterns that EBSeq uses to classify genes are based on the expression level in each condition. For example, genes with the same expression level in all conditions are classified as Pattern 1. Genes that show differential expression between OPCs and Schwann cells regardless of electrical stimulation but have the same level of expression in cells of the same type are classified as Pattern 4. Similarly, genes that are differentially expressed between EF-stimulated and control cells but have the same level regardless of cell type are classified as Pattern 6. However, there are several other patterns that include genes that show differential expression between EF-stimulated and control cells, but do not have the same expression level in both types of EF-stimulated or control cells (Patterns 10–13). The patterns reported by EBSeq also do not differentiate between the up-regulation and down-regulation of genes. Since we were interested in the direction of differential expression after the EF, regardless of the initial level of expression in each particular cell type, we manually combined genes differentially expressed genes (FDR < 0.05) from multiple RSEM patterns into a new set of more informative patterns. (Supplemental Table 1).

Gene ontology functional enrichment analysis and signaling pathway analysis of the significantly changed genes were performed using the Database for Annotation, Visualization, and Integrated Discovery (DAVID)^67^.

## Supplementary information

Supplementary Information.

Supplementary Table 1.

Supplementary Table 2.

## Data Availability

All data generated or analysed during this study are included in this published article and its supplementary information files.
