# Supplementary material for: Genetic analysis of the molecular regulation of electric fields-guided glia migration
Source: Sci Rep. 2020 Oct 8;10:16821. doi: 10.1038/s41598-020-74085-x (PMC7546725; doi:10.1038/s41598-020-74085-x)
Supplement: Supplementary file 1 — Supplementary Information. [file 41598_2020_74085_MOESM1_ESM.docx]

**Genetic analysis of the molecular regulation of electric fields-guided glia migration**

Li Yao^1*^, Teresa Shippy^2^, Yongchao Li^1^

1 Department of Biological Sciences, Wichita State University, 1845 Fairmount Street, Wichita, KS 67260

2 Bioinformatics Specialist, KSU Bioinformatics Center, Kansas State University, Manhattan, KS. 66506

*Corresponding author:

Li Yao

Department of Biological Sciences, Wichita State University, Wichita, KS

Phone: (316)9786766; Fax: 316-978-3772; Email: [li.yao@wichita.edu](mailto:li.yao@wichita.edu)
