# Supplementary material for: Genetic analysis of the molecular regulation of electric fields-guided glia migration
Source: Sci Rep. 2020 Oct 8;10:16821. doi: 10.1038/s41598-020-74085-x (PMC7546725; doi:10.1038/s41598-020-74085-x)
Supplement: Supplementary file 2 — Supplementary Table 1. [file 41598_2020_74085_MOESM2_ESM.docx]

Supplemental table 1. Differential expression patterns classified by direction of expression change. For each pattern, the number of genes meeting the 5 percent FDR cutoff and the RSEM expression patterns these genes originated from are also shown.

| **Differential Expression Pattern** | **# of genes** | **RSEM Patterns** |
| --- | --- | --- |
| Both Down | 193 | 6, 11, 12, 13, 15 |
| Both Up | 154 | 6, 11, 12, 13, 15 |
| SC-Down/OPC No change | 313 | 2, 3, 9 |
| SC-Up/OPC No change | 410 | 2, 3, 9 |
| OPC-Down/SC No change | 402 | 5, 8, 14 |
| OPC-Up/SC No change | 341 | 5, 8, 14 |
| SC-Up/OPC-Down | 52 | 7, 10, 11, 12, 13, 15 |
| OPC-Up/SC-Down | 42 | 10, 11, 12, 13, 15 |
