# Supplementary material for: Genetic analysis of the molecular regulation of electric fields-guided glia migration
Source: Sci Rep. 2020 Oct 8;10:16821. doi: 10.1038/s41598-020-74085-x (PMC7546725; doi:10.1038/s41598-020-74085-x)
Supplement: Supplementary file 3 — Supplementary Table 2. [file 41598_2020_74085_MOESM3_ESM.docx]

Supplemental table 2. Definitions and Counts of EBSeq expression patterns. For each gene, expression levels (1-4) are assigned in the following order: OPC EF, OPC Ctrl, SC EF, SC Ctrl. Different expression levels do not indicate the direction of difference. Gene counts for each pattern are shown for all genes as well as for FDR cutoffs of 0.05 and 0.01.

| Pattern | OPC EF | OPC Ctrl | SC EF | SC Ctrl | All genes | FDR < 0.05 | FDR < 0.01 |
| --- | --- | --- | --- | --- | --- | --- | --- |
| 1 (nonDE) | 1 | 1 | 1 | 1 | 10949 |  |  |
| 2 | 1 | 1 | 1 | 2 | 88 | 68 | 58 |
| 3 | 1 | 1 | 2 | 1 | 39 | 32 | 29 |
| 4 | 1 | 1 | 2 | 2 | 8999 | 7534 | 6961 |
| 5 | 1 | 2 | 1 | 1 | 178 | 132 | 110 |
| 6 | 1 | 2 | 1 | 2 | 132 | 92 | 76 |
| 7 | 1 | 2 | 2 | 1 | 5 | 2 | 2 |
| 8 | 1 | 2 | 2 | 2 | 162 | 118 | 90 |
| 9 | 1 | 1 | 2 | 3 | 602 | 600 | 599 |
| 10 | 1 | 2 | 1 | 3 | 2 | 2 | 2 |
| 11 | 1 | 2 | 2 | 3 | 31 | 31 | 31 |
| 12 | 1 | 2 | 3 | 1 | 54 | 54 | 54 |
| 13 | 1 | 2 | 3 | 2 | 10 | 10 | 10 |
| 14 | 1 | 2 | 3 | 3 | 460 | 454 | 453 |
| 15 | 1 | 2 | 3 | 4 | 235 | 235 | 235 |
| Totals |  |  |  |  | 21946 | 9364 | 8710 |
